# ﻿A new species of the genus *Hebius* (Squamata, Natricidae), previously confused with *H. boulengeri* (Gressitt, 1937)

**DOI:** 10.3897/zookeys.1254.161130

**Published:** 2025-10-03

**Authors:** Shuo Liu, JiShan Wang, Mian Hou, Liang Zhang, Qiaoyan Wang, Chunmiao Zong, Jiang Zhou, Dingqi Rao, Patrick David, Gernot Vogel

**Affiliations:** 1 Kunming Natural History Museum of Zoology, Kunming Institute of Zoology, Chinese Academy of Sciences, Kunming, Yunnan 650223, China; 2 Yunnan Key Laboratory of Biodiversity Information, Kunming Institute of Zoology, Chinese Academy of Sciences, Kunming, Yunnan 650201, China; 3 Southwest Survey and Planning Institute of National Forestry and Grassland Administration, Kunming, Yunnan 650031, China; 4 College of Continuing (Online) Education, Sichuan Normal University, Chengdu, Sichuan 610066, China; 5 Guangdong Key Laboratory of Animal Conservation and Resource Utilization, Guangdong Public Laboratory of Wild Animal Conservation and Utilization, Institute of Zoology, Guangdong Academy of Sciences, Guangzhou, Guangdong 510260, China; 6 Research Institute of Yunnan Xishuangbanna National Nature Reserve, Jinghong, Yunnan 666100, China; 7 Management and Protection Bureau of Mojiang Xiqi Alsophila Provincial Nature Reserve, Mojiang, Yunnan 654800, China; 8 Kunming Institute of Zoology, Chinese Academy of Sciences, Kunming, Yunnan 650201, China; 9 Reptiles & Amphibiens, Institut de Systématique, Évolution et Biodiversité (ISYEB), Muséum National d’Histoire Naturelle, CP 30, 57 rue Cuvier, F-75005 Paris, France; 10 Society for South East Asian Herpetology, Im Sand-3, D-69115 Heidelberg, Germany

**Keywords:** Keelback snake, mitochondrial gene, morphological comparison, phylogenetic analysis, Pu’er City, taxonomy, Xishuangbanna Prefecture

## Abstract

A new species of the genus *Hebius* is described from Yunnan Province, China. The new species was previously identified as *H. boulengeri*, but it can be separated from *H. boulengeri* and other species of this genus by a combination of the following characters: 19-19-17 dorsal scale rows, 148–155 ventrals, 90–105 subcaudals, one preocular, three postoculars, 23–25 maxillary teeth, a distinct continuous white streak from posteroventral margin of eye extending backward and upward to nape to connect with dorsolateral stripe on each side, and white venter. The genetic divergence between the new species and *H. boulengeri* was 6.6% and the genetic divergences between the new species and other congeners ranged from 6.8% to 15.9% in the mitochondrial cytochrome b gene.

## ﻿Introduction

The genus *Hebius* Thompson, 1913 is the most speciose genus in the family Natricidae (Bohra et al. 2025; Uetz et al. 2025). It is a group of small to medium-sized, non-venomous, semi-aquatic snakes that are widely distributed throughout southern and southeastern Asia (Guo et al. 2014; Liu et al. 2018; Purkayastha and David 2019; Deepak et al. 2022; Hauser et al. 2022; Ma et al. 2023; Xu et al. 2023, 2024; Bohra et al. 2025). Due to interspecific morphological similarities, the species of this genus often cannot be accurately identified by morphology, which leads to the species diversity of this genus being far underestimated (Liu et al. 2018; Ren et al. 2018; Xu et al. 2023).

Boulenger’s keelback, *H. boulengeri* (Gressitt, 1937), was originally described as *Natrix boulengeri* by Gressitt (1937) from Tai-yong, Eastern Kwangtung, China (Jieyang City, Guangdong Province, China). Subsequently, this species was transferred to the genus *Amphiesma* and later to the genus *Hebius* (Guo et al. 2014). Previously, this species was considered to be widely distributed in southern China, including Guangdong, Fujian, Jiangxi, Hunan, Hainan, Guizhou, and Yunnan provinces, Chongqing Municipality, Guangxi Autonomous Region, and Hong Kong Special Administrative Region, as well as Vietnam and Cambodia (Huang 2021; Wang 2021; Uetz et al. 2025). In Yunnan Province, *H. boulengeri* was recorded from Honghe, Xishuangbanna, Dehong, Nujiang, and Diqing prefectures, as well as Pu’er, Lincang, and Baoshan cities (Zhao et al. 1998; Yang and Rao 2008).

During our fieldwork in Yunnan Province, China, from 2018 to 2023, four specimens superficially resembling *H. boulengeri* were collected from Xishuangbanna Prefecture and Pu’er City. Morphological comparison and molecular data analysis showed that these specimens are distinct from *H. boulengeri* and other species of the genus. Herein, we describe a new species of the genus *Hebius* based on these four specimens.

## ﻿Material and methods

The specimens were collected by hand and stored in 75% ethanol after being photographed, and then deposited at Kunming Natural History Museum of Zoology, Kunming Institute of Zoology, Chinese Academy of Sciences (KIZ).

Measurements were taken using digital calipers to the nearest 0.1 mm. Bilateral scale counts were given as left/right. The abbreviations of measurements and scale counts are as follows:

**ATem** number of anterior temporals

**DLip** distance between the lower edge of the lip and the lower margin of the eye

**DS** numbers of dorsal scale rows behind the neck, at the midbody, and before the vent, respectively

**EDH** horizontal diameter of the eye

**EDV** vertical diameter of the eye

**HL** head length

**IL** number of infralabials

**MXT** number of maxillary teeth

**PosOc** number of postoculars

**PreOc** number of preoculars

**Sc** number of subcaudals

**SL** number of supralabials

**SnL** snout length, between the eye and the anterior margin of the rostral

**SVL** snout–vent length

**TaL** tail length

**TL** total length

**Ven** number of ventrals

We examined 81 specimens of *H. boulengeri* from various museums, including the holotype and paratype. The morphological data of other species of this genus were obtained from literature (Gressitt 1937; Zhao et al. 1998; Yang and Rao 2008; Liu et al. 2018; Ren et al. 2018; Purkayastha and David 2019; Huang 2021; Hauser et al. 2022; Ma et al. 2023; Xu et al. 2023, 2024; Bohra et al. 2025).

Total Genomic DNA for the four newly collected specimens was extracted from the liver tissue. A fragment of the mitochondrial cytochrome b gene (cytb) was amplified using the primer pairs L14910: 5′-GACCTGTGATMTGAAAAACCAYCGTTGT-3′ and H16064: 5′-CTTTGGTTTACAAGAACAATGCTTTA-3′ (Burbrink et al. 2000). The amplification products were purified and sequenced by Sangon Biotech (Shanghai) Co., Ltd. The generated sequences were assembled using SeqMan in Lasergene 7.1 (Burland 2000). *Amphiesma stolatum* (Linnaeus, 1758) was used as outgroup according to Bohra et al. (2025). The sequences of 34 species of the genus *Hebius* and the outgroup were obtained from GenBank (Table 1).

**Table 1.** Sequences of *Hebius* species and outgroup used in this study.

Species name	locality	Voucher	Accession	Reference
*H. shantianfangi* sp. nov.	Mengla, Yunnan, China	KIZ 064970	PX206432	This study
*H. shantianfangi* sp. nov.	Pu’er, Yunnan, China	KIZ 064971	PX206433	This study
*H. shantianfangi* sp. nov.	Mojiang, Yunnan, China	KIZ 064972	PX206434	This study
*H. shantianfangi* sp. nov.	Mojiang, Yunnan, China	KIZ 064973	PX206435	This study
*H. andreae*	Khammouane, Laos	VNUF R.2017.25	MK253674	Ziegler et al. 2019
*H. annamensis*	Xe Kong, Laos	FMNH 258637	OK315812	Deepak et al. 2021
*H. atemporalis*	Pu Hoat, Nge An, Vietnam	ZMMU NAP-07877	OK315813	Deepak et al. 2021
*H. bitaeniatus*	Chiang Mai, Thailand	AUP-00062	OK315816	Deepak et al. 2021
*H. boulengeri*	Ha Giang, Vietnam	AMNH 148562	KJ685664	Guo et al. 2014
*H. boulengeri*	Tam Dao, Vinh Phuc, Vietnam	ZMMU NAP-08675	OK315817	Deepak et al. 2021
*H. boulengeri*	Hechi, Guagnxi, China	SYS r001552	MK201520	Li et al. 2020
*H. boulengeri*	Fujian, China	GP2433	KJ685699	Guo et al. 2014
*H. boulengeri*	Guangdong, China	GP1789	KJ685684	Guo et al. 2014
*H. boulengeri*	Chebaling, Guangdong, China	RE55	MK201380	Li et al. 2020
*H. boulengeri*	Jinpenshan, Jiangxi, China	SYS r001371	MK201483	Li et al. 2020
*H. boulengeri*	Hainan, China	GP2134	KJ685691	Guo et al. 2014
*H. boulengeri*	Hainan, China	MVZ 236752	KJ685710	Guo et al. 2014
*H. boulengeri*	Hainan, China	HS11107	MK201295	Li et al. 2020
*H. boulengeri*	Dawuling, Guangdong, China	SYS r001506	MK201509	Li et al. 2020
*H. chapaensis*	Lao Cai, Vietnam	VNMN 06103	MH778700	Ren et al. 2018
*H. citrinoventer*	Yingjiang, Yunnan, China	ANU 20230016	PP472750	Xu et al. 2024
*H. clerki*	Nujiang, Yunnan, China	CAS 215036	KJ685666	Guo et al. 2014
*H. concelarus*	Miyakojima, Okinawa, Japan	KUZ:R20253	AB989268	Kaito and Mamoru 2016
*H. craspedogaster*	Guizhou, China	GP1240	KJ685672	Guo et al. 2014
*H. deschauenseei*	Chiang Mai, Thailand	AUP-00182	OK315827	Deepak et al. 2021
*H. gilhodesi*	Kachin state, Myanmar	CAS 221504	KJ685668	Guo et al. 2014
*H. gilhodesi*	Kachin state, Myanmar	CAS 221525	KJ685669	Guo et al. 2014
*H. igneus*	Ha Giang, Vietnam	AMNH 148575	KJ685665	Guo et al. 2014
*H. ishigakiensis*	Iriomotejima, Okinawa, Japan	KUZ:R33043	AB989292	Kaito and Mamoru 2016
*H. jingdongensis*	Jingdong, Yunnan, China	CIB 119044	OR285310	Ma et al. 2023
*H. johannis*	Yunnan, China	KIZ 014484	MZ570479	Hou et al. 2021
*H. khasiensis*	Mairang, Meghalaya, India	MZMU 3526	PQ288047	Bohra et al. 2025
*H. khasiensis*	Sailam, Aizawl, Mizoram, India	MZMU 2540	PQ288048	Bohra et al. 2025
*H. maximus*	Sichuan, China	GP864	KJ685706	Guo et al. 2014
*H. metusia*	Sichuan, China	GP871	KJ685707	Guo et al. 2014
*H. modestus*	Yunnan, China	CAS 234262	KJ685671	Guo et al. 2014
*H. octolineatus*	Yunnan, China	GP1569	KJ685678	Guo et al. 2014
*H. optatus*	Guizhou, China	GP1885	KJ685687	Guo et al. 2014
*H. popei*	Hainan, China	GP2169	KJ685692	Guo et al. 2014
*H. pryeri*	Tokunoshima, Kagoshima, Japan	KUZ:R34062	AB989126	Kaito and Mamoru 2016
*H. sangzhiensis*	Hunan, China	SYNU 08070350	MK340763	Zhou et al. 2019
*H. sauteri*	Taiwan, China	GP2549	KJ685701	Guo et al. 2014
*H. septemlineatus*	Tengchong, Yunnan, China	KIZ 037720	MZ570486	Hou et al. 2021
*H. taronensis*	Kachin State, Myanmar	CAS 224426	OK315828	Deepak et al. 2021
*H. venningi*	Kachin, Myanmar	CAS 233206	KJ685670	Guo et al. 2014
*H. vibakari*	Kyoto, Japan	KUZ:R21587	AB989302	Kaito and Mamoru 2016
*H. weixiensis*	Weixi, Yunnan, China	KIZ 035740	MZ570488	Hou et al. 2021
*H. yanbianensis*	Yanbian, Sichuan, China	GP4006	MH532291	Guo et al. 2014
*H. youjiangensis*	Baise, Guangxi, China	HSR22184	OQ085073	Xu et al. 2023
*H.* sp.	Quang Nam, Vietnam	AMNH 148552	KJ685663	Guo et al. 2014
*H.* sp.	Kon Ka Kinh, Gia Lai, Vietnam	ZMMU R-16531	OK315818	Deepak et al. 2021
*H.* sp.	Vietnam	HT0679	LC325327	Takeuchi et al. 2018
*Amphiesma stolatum*	Ha Giang, Vietnam	CAS 215037	KJ685667	Guo et al. 2014

Sequences were aligned using MAFFT 7.471 (Katoh and Standley 2013) with default parameters. The genetic divergence (uncorrected *p*-distance) between species was calculated in MEGA 12.0.10 (Kumar et al. 2024). Bayesian-inference (BI) and maximum-likelihood analysis (ML) were used to construct the phylogenetic tree. The best-fit substitution models for BI and ML, respectively, were chosen using the Akaike information criterion in ModelFinder (Kalyaanamoorthy et al. 2017). The BI was performed in MrBayes 3.2.6 (Ronquist et al. 2012) using the model GTR+F+I+G4, and the Markov chains were run for one million generations with sampled every 100 generations. The ML was performed in IQ-TREE 1.6.12 (Nguyen et al. 2015) using the model TIM2+F+I+G4, and nodal support was estimated by 1,000 ultrafast bootstrap replicates.

## ﻿Results

**Figure 1.**
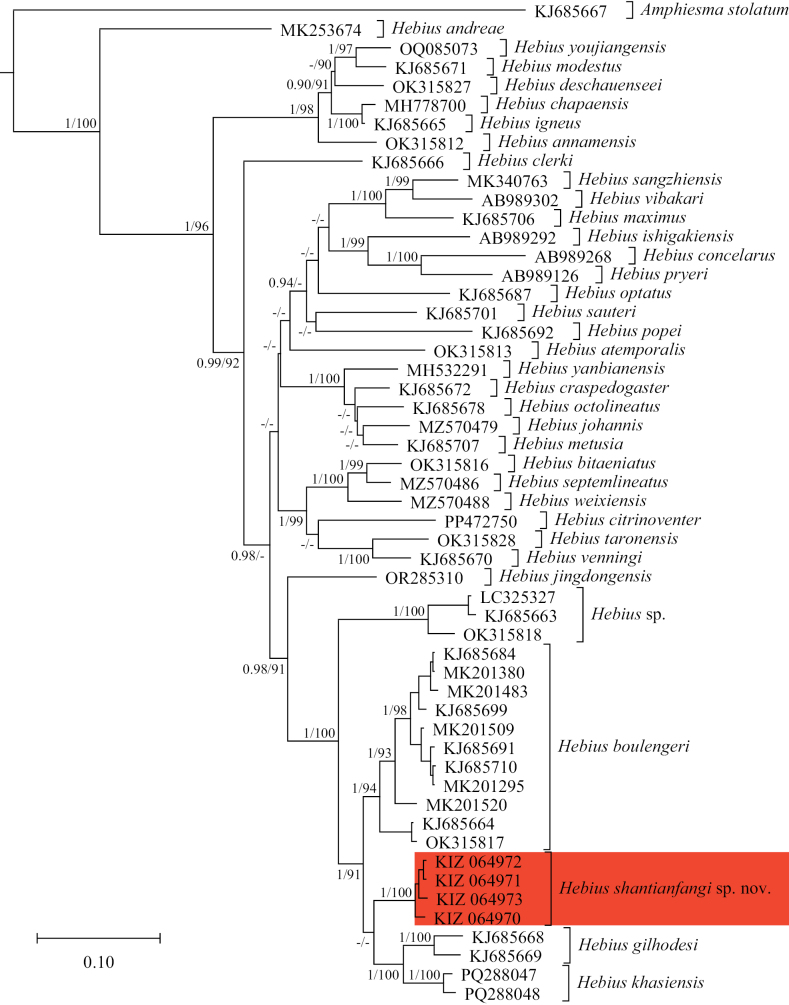
Bayesian phylogenetic tree based on cytb sequences. The numbers after and behind “/” indicate the Bayesian posterior probabilities (above 0.90 retained) and maximum likelihood ultrafast bootstrap values (above 90 retained), respectively.

The phylogenetic trees resulted in very similar topologies by BI and ML. The sequences of the newly collected specimens from Yunnan clustered together to form a distinct lineage between the clade of *H. boulengeri* and the clade consisting of *H. gilhodesi* (Wall, 1925) and *H. khasiensis* (Boulenger, 1890), but the exact phylogenetic position of the novel lineage was not resolved (Fig. 1). The genetic divergence between the novel lineage and *H. boulengeri* was 6.6%, the genetic divergence between the novel lineage and *H. khasiensis* was 6.8%, and the genetic divergence between the novel lineage and *H. gilhodesi* was 7.6% (Table 2).

**Table 2.** Percentage genetic divergence (uncorrected *p*-distance) based on cytb sequences between species of *Hebius*. Values between the new species and closely related species are highlighted in bold.

	1	2	3	4	5	6	7	8	9	10	11	12	13	14	15	16	17
1 *Hebius shantianfangi* sp. nov.																	
2 *Hebius andreae*	15.9																
3 *Hebius annamensis*	14.0	14.8															
4 *Hebius atemporalis*	11.8	16.3	14.5														
5 *Hebius bitaeniatus*	11.9	15.4	13.9	10.9													
6 *Hebius boulengeri*	**6.6**	16.2	13.0	12.0	10.8												
7 *Hebius chapaensis*	12.9	14.2	6.3	13.1	13.6	12.4											
8 *Hebius citrinoventer*	13.3	16.2	14.2	12.9	10.5	13.1	14.1										
9 *Hebius clerki*	11.2	15.7	13.5	12.3	12.3	10.7	12.3	12.9									
10 *Hebius concelarus*	13.6	18.0	16.1	14.4	13.2	13.7	15.4	13.8	14.0								
11 *Hebius craspedogaster*	11.3	16.2	13.2	11.4	11.8	10.8	13.0	12.0	12.0	14.0							
12 *Hebius deschauenseei*	13.2	15.4	6.9	14.3	13.6	13.2	6.1	14.5	13.3	15.4	13.2						
13 *Hebius gilhodesi*	**7.6**	16.6	14.4	12.4	12.3	7.8	13.3	14.3	11.7	13.4	12.2	13.7					
14 *Hebius igneus*	13.9	15.3	6.0	13.4	13.3	13.4	1.0	14.4	13.1	15.2	13.0	5.2	14.0				
15 *Hebius ishigakiensis*	12.4	17.0	14.2	12.6	11.7	12.2	14.1	12.9	11.9	12.1	12.4	14.4	13.1	14.1			
16 *Hebius jingdongensis*	10.5	14.8	12.6	11.0	11.2	9.6	12.5	11.7	12.3	14.0	10.4	11.9	11.2	12.4	12.7		
17 *Hebius johannis*	10.0	14.2	12.0	11.0	10.7	10.0	11.9	11.1	11.3	12.5	5.0	12.4	11.5	12.3	12.3	10.4	
18 *Hebius khasiensis*	**6.8**	16.0	13.3	12.5	12.7	7.5	12.0	13.3	11.8	14.0	11.9	12.7	6.2	12.6	13.1	10.1	10.9
19 *Hebius maximus*	13.1	14.5	13.7	13.1	10.8	13.4	13.2	13.6	12.3	13.5	11.8	12.5	13.8	13.2	11.2	12.8	11.7
20 *Hebius metusia*	10.9	15.2	13.1	10.6	10.4	10.6	12.5	11.8	12.1	13.3	4.6	12.5	12.0	12.7	12.2	9.8	4.7
21 *Hebius modestus*	14.7	15.6	7.0	15.0	13.5	14.1	5.6	14.5	13.1	16.0	14.4	6.1	15.0	5.1	13.7	12.8	13.2
22 *Hebius octolineatus*	11.5	14.7	12.9	11.0	11.6	10.6	12.2	11.8	11.8	12.8	5.1	12.7	12.2	12.8	12.1	10.1	5.3
23 *Hebius optatus*	13.2	17.1	15.5	13.1	12.0	13.0	14.1	13.7	13.4	12.8	12.4	15.1	13.7	14.8	11.8	13.2	12.0
24 *Hebius popei*	12.3	16.8	14.8	13.1	12.3	13.3	14.5	14.4	12.7	14.4	13.4	14.5	13.6	15.3	12.4	12.0	12.4
25 *Hebius pryeri*	12.8	17.3	15.7	12.8	12.8	12.8	15.5	13.0	12.7	9.1	13.1	15.8	14.1	15.5	10.4	13.3	12.3
26 *Hebius sangzhiensis*	12.9	15.3	14.5	13.6	10.8	12.6	14.0	13.4	13.0	12.0	11.8	13.3	12.8	14.1	11.1	12.8	10.9
27 *Hebius sauteri*	11.9	16.7	14.4	12.1	11.2	12.4	13.8	12.0	12.0	12.9	10.7	14.2	12.2	13.9	10.7	10.2	11.0
28 *Hebius septemlineatus*	11.3	15.3	14.0	11.6	3.5	10.9	13.8	10.5	12.1	12.7	11.2	13.2	12.0	13.7	11.5	10.6	10.5
29 *Hebius taronensis*	11.4	15.4	14.7	13.0	10.1	13.0	13.5	10.8	12.7	13.8	11.9	13.5	13.6	14.3	12.4	12.0	11.0
30 *Hebius venningi*	11.6	14.1	14.2	11.8	8.8	12.3	12.2	9.7	12.0	13.0	11.0	13.5	12.8	13.1	12.1	11.1	9.8
31 *Hebius vibakari*	13.0	14.2	13.9	12.3	10.5	12.3	13.7	13.4	13.3	13.2	12.1	13.8	13.3	13.5	12.0	11.6	11.5
32 *Hebius weixiensis*	11.6	15.3	13.3	10.9	6.4	10.6	12.7	10.8	11.0	12.7	10.1	13.5	12.0	12.9	12.1	10.2	10.5
33 *Hebius yanbianensis*	11.5	15.4	13.7	11.2	11.2	10.8	13.1	11.8	11.9	13.3	5.1	13.2	12.1	13.3	12.1	11.0	6.1
34 *Hebius youjiangensis*	14.4	15.8	7.3	14.3	14.2	14.0	5.6	14.6	12.4	15.5	13.9	5.9	14.7	5.4	14.2	12.7	13.0
35 *Hebius* sp.	9.3	15.3	14.4	13.0	12.6	9.0	13.3	13.4	11.6	13.8	11.9	13.8	10.6	13.8	12.4	11.1	11.0
	18	19	20	21	22	23	24	25	26	27	28	29	30	31	32	33	34
19 *Hebius maximus*	14.0																
20 *Hebius metusia*	11.2	11.8															
21 *Hebius modestus*	13.2	13.0	14.2														
22 *Hebius octolineatus*	11.1	12.0	4.9	13.9													
23 *Hebius optatus*	14.7	11.6	12.3	14.9	12.8												
24 *Hebius popei*	13.0	12.2	12.8	14.3	13.0	11.6											
25 *Hebius pryeri*	14.0	12.1	12.0	15.3	11.8	12.9	13.7										
26 *Hebius sangzhiensis*	13.3	7.6	12.3	13.7	11.5	12.0	12.8	11.1									
27 *Hebius sauteri*	12.9	11.2	10.8	14.2	11.5	11.4	11.6	11.8	11.5								
28 *Hebius septemlineatus*	12.3	10.4	10.2	13.8	10.6	11.8	11.5	12.3	10.4	10.7							
29 *Hebius taronensis*	12.9	11.4	11.4	14.1	12.0	13.2	12.2	13.0	12.3	12.2	9.5						
30 *Hebius venningi*	12.7	11.6	10.1	13.4	10.9	12.3	11.8	12.1	12.1	11.8	9.2	5.6					
31 *Hebius vibakari*	13.4	8.2	11.6	13.5	11.6	11.1	12.8	12.4	6.1	11.2	10.0	11.5	11.2				
32 *Hebius weixiensis*	11.9	11.1	9.8	13.5	10.4	11.5	12.8	12.5	10.4	10.7	5.7	10.3	9.2	10.3			
33 *Hebius yanbianensis*	11.7	11.5	5.4	14.2	6.4	12.4	13.0	12.4	11.4	11.5	10.6	11.7	10.5	11.7	9.3		
34 *Hebius youjiangensis*	13.5	13.4	13.9	4.1	13.5	15.2	14.7	15.7	13.9	13.9	14.2	14.6	13.8	13.7	13.5	14.5	
35 *Hebius* sp.	10.2	12.9	11.8	14.5	11.1	13.9	14.7	13.1	13.4	13.1	12.9	13.2	12.6	13.1	12.7	12.4	14.3

Morphologically, the newly collected specimens from Yunnan can be distinguished from all named species of the genus. The newly collected specimens differ from all other species of the genus, except *H. boulengeri*, in having a distinct continuous white streak from the posteroventral margin of the eye extending backward and upward to the nape on each side. The newly collected specimens differ from *H. boulengeri* in having fewer maxillary teeth (Suppl. material 1).

Based on the results of molecular and morphological analysis, the newly collected specimens from Yunnan should be regarded as a distinct new species, which we formally describe here.

### ﻿Taxonomic accounts

#### ﻿ Hebius shantianfangi sp. nov.

Taxon classificationAnimaliaSquamataNatricidae

32DE4984-247A-5405-8CE1-ECE5CB5D0D9A

https://zoobank.org/B7CE0216-9A4B-42B6-9AFA-40BDB92CBA73

**Figure 2.**
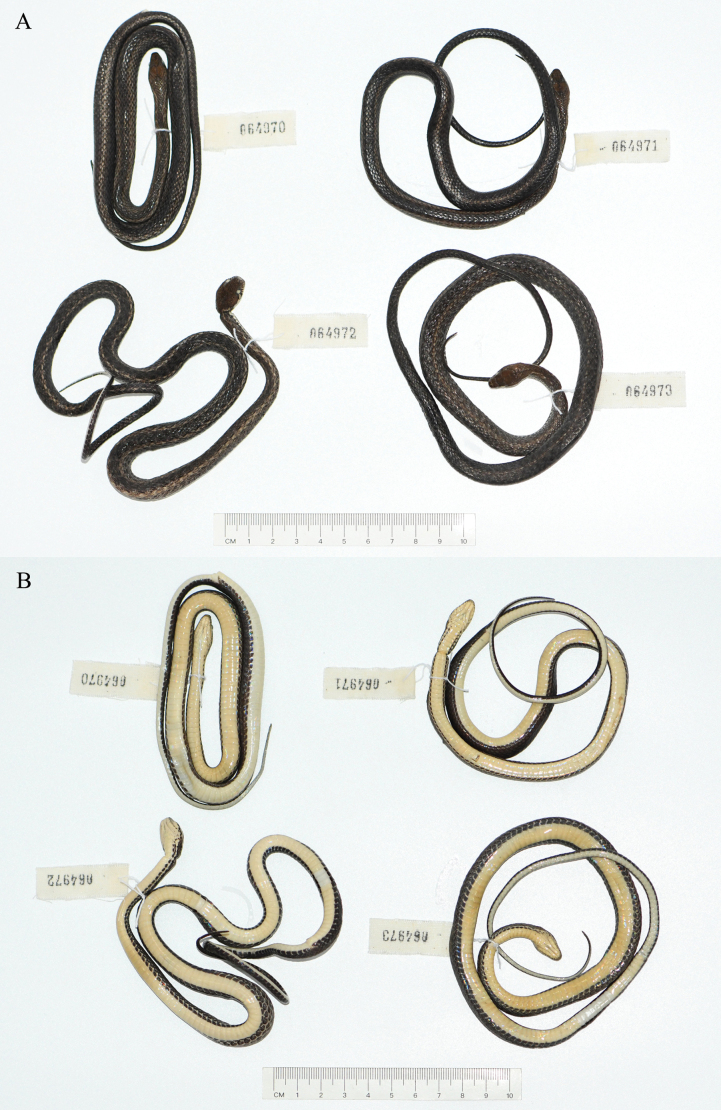
The type specimens of *Hebius shantianfangi* sp. nov. in preservative. A. Dorsal view; B. Ventral view.

**Figure 3.**
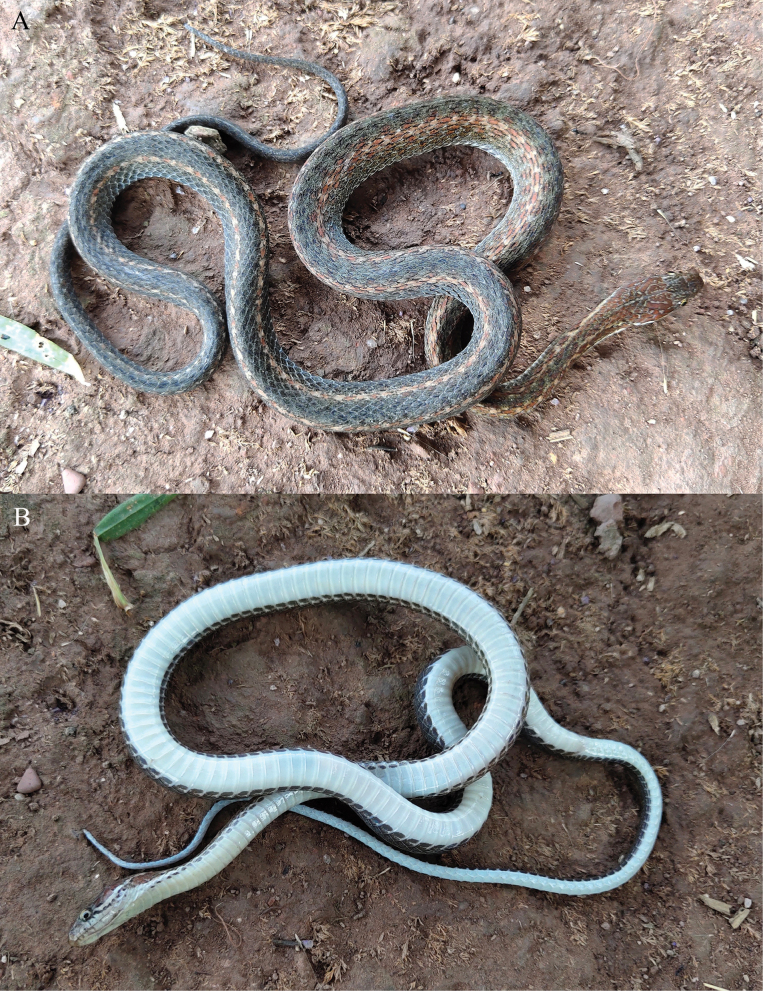
The holotype (KIZ 064970) of *Hebius shantianfangi* sp. nov. in life. A. Dorsal view; B. Ventral view.

**Figure 4.**
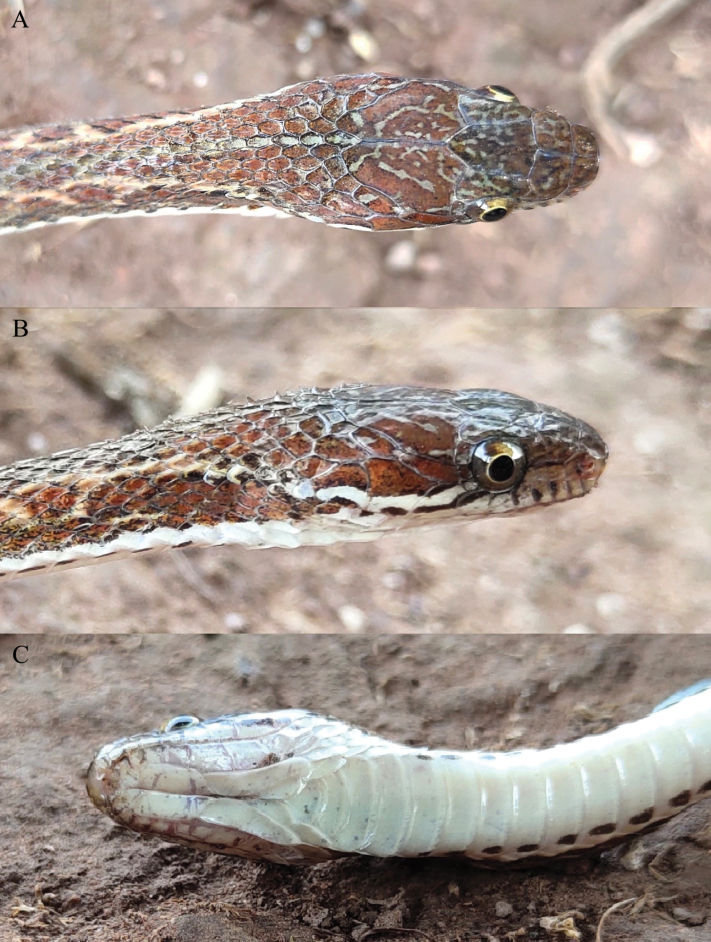
Close-up views of the head of the holotype (KIZ 064970) of *Hebius shantianfangi* sp. nov. in life. A. Dorsal view; B. Lateral view; C. Ventral view.

**Figure 5.**
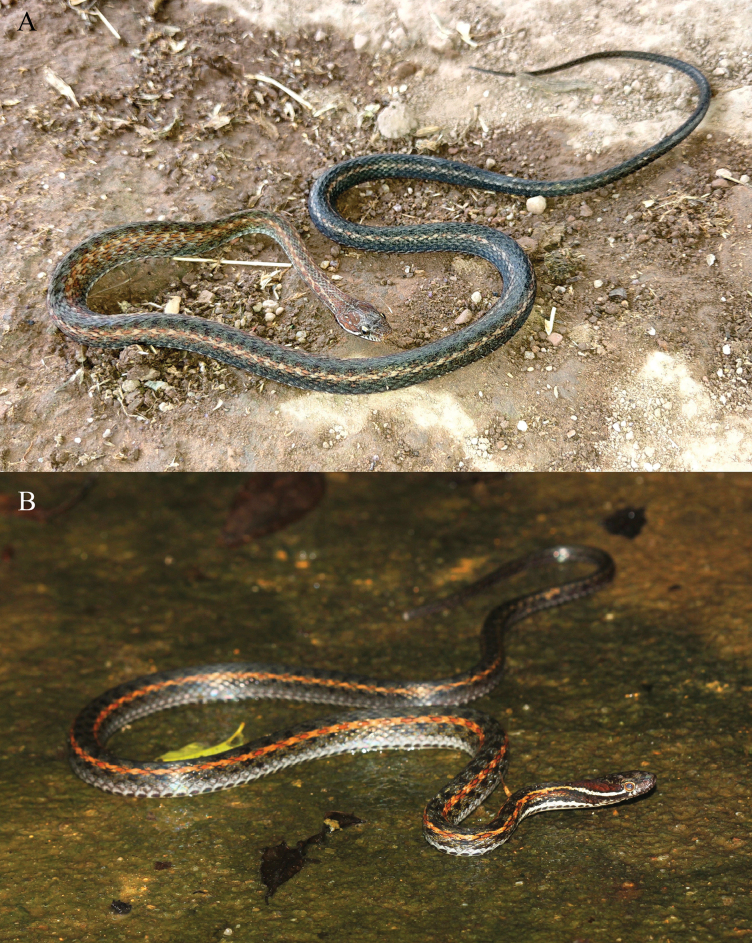
Comparison of the body coloration between *Hebius shantianfangi* sp. nov. and *H. boulengeri*. A. Holotype (KIZ 064970) of *Hebius shantianfangi* sp. nov.; B. *H. boulengeri* from Shaoguan City, Guangdong Province, China.

**Figure 6.**
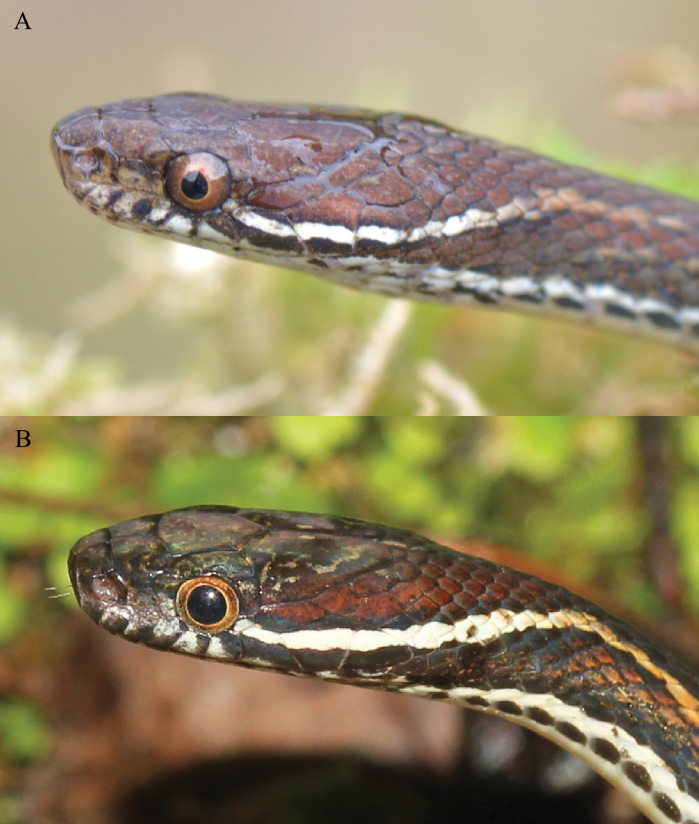
Comparison of the lateral side of the head between *Hebius shantianfangi* sp. nov. and *H. boulengeri*. A. Paratype (KIZ 064973) of *Hebius shantianfangi* sp. nov.; B. *H. boulengeri* from Shaoguan City, Guangdong Province, China.

Figs 2, 3, 4, 5A, 6A

##### Type materials.

***Holotype*** • KIZ 064970; adult female; Longlin Village, Mengla Town, Mengla County, Xishuangbanna Prefecture, Yunnan Province, China; 21°38′15″N, 101°27′0″E, 1,040 m; 27 April 2019; JiShan Wang leg. ***Paratypes*** • KIZ 064971; adult male; Yutang Village, Yixiang Town, Simao District, Pu’er City, Yunnan Province, China; 22°36′58″N, 101°7′32″E, 1,300 m; 18 September 2018; Mian Hou leg. • KIZ 064972; adult female; Guangfeng Village, Sinanjiang Town, Mojiang County, Pu’er City, Yunnan Province, China; 22°56′49″N, 101°49′51″E, 1810 m; 31 May 2023; Mo Wang leg. • KIZ 064973; adult female; Bingli Village, Yutang Town, Mojiang County, Pu’er City, Yunnan Province, China; 23°7′10″N, 101°31′46″E, 1850 m; 26 June 2023; Mo Wang leg.

##### Diagnosis.

Body slender, SVL 332–406 mm, TL 506–610 mm; tail relatively long, TaL/TL 0.31–0.34; dorsal scales in 19-19-17 rows, all strongly keeled except for 1^st^ row on each side; ventrals 148–155; cloacal plate divided; subcaudals 90–105, paired; loreal one, not entering orbit; preocular one, postoculars three; supralabials mostly nine, rarely ten, mostly 4^th^–6^th^ entering orbit, rarely 5^th^–7^th^ entering orbit; infralabials 9–11; maxillary teeth 23–25, last one or two distinctly enlarged, no diastema separating enlarged posterior teeth with anterior ones; a distinct continuous white streak from posteroventral margin of eye extending backward and upward to nape on each side; dorsolateral stripes present; venter white, ventrolateral blotches present, composed of a dark grey spot on edge of each ventral on each side.

##### Etymology.

Named after the late renowned Chinese storytelling artist, Tianfang Shan (17.12.1934–11.09.2018). This species was previously confused with *H. boulengeri*, whose Chinese name is “白眉腹链蛇”. In China, when “白眉” is mentioned, people first think of Tianfang Shan’s classic storytelling “白眉大侠”. This specific epithet commemorates Tianfang Shan, who devoted his whole life to storytelling and left countless indelible memories for people. We suggest “Shan’s keelback” as the English name and “单氏腹链蛇” (Pinyin: shàn shì fù liàn shé) as the Chinese name of the new species.

##### Description of holotype.

Body cylindrical, slender (TL 539 mm), tail relatively long (TaL/TL 0.31); head moderately large, elongate (HL/SVL 0.044), distinct from neck; snout narrow, relatively long (SnL/HL 0.24), tip obtuse; eye large (EDH/SnL 0.72, EDV/DLip 2.36), pupils round; nostrils oriented laterally.

Rostral wider than high, approximately twice as width as high, nearly invisible from above; nasal divided into two halves, posterior half slightly larger than anterior half; internasals two; prefrontals two, in contact with loreal, prefrontal suture slightly longer than internasal suture; frontal shield shaped, elongate, approximately 1.4 times as long as width, approximately 2.2 times length of prefrontal suture; supraocular one on each side, elongate, almost as long as frontal; parietals two, large, approximately 1.9 times as long as width, almost 1.5 times length of frontal, parietal suture almost equal to length of frontal; loreal one on each side, in contact with 2^nd^–3^rd^ supralabials on left side and 3^rd^–4^th^ supralabials on right side, not entering orbit; preoculars one on each side, vertically elongate; postoculars three on each side, size decreasing from top to bottom; supralabials nine on left side and ten on right side, 4^th^–6^th^ entering orbit on left side and 5^th^–7^th^ entering orbit on right side, penultimate one largest; anterior temporal one on each side, posterior temporals two on each side; mental triangular, small; infralabials ten on each side, first pair in contact with each other after mental, 1^st^–5^th^ in contact with anterior chin shield; chin shield two pairs; posterior chin shields longer than anterior ones.

Dorsal scales in 19-19-17 rows, all strongly keeled except for 1^st^ row on each side smooth or feebly keeled; preventrals two, ventrals 148; subcaudals 90, paired; cloacal plate divided.

Maxillary teeth 25 (24+1), becoming enlarged posteriorly with last one distinctly enlarged, no proper diastema between last enlarged one and anterior ones.

##### Coloration in life.

Dorsal surface of head reddish brown with some white vermiculate stripes or spots; lateral surface of head reddish brown, a distinct continuous white streak from posteroventral margin of eye extending backward and upward to nape to connect with dorsolateral stripe on each side, edges of white streak jagged and bordered by indistinct black color; upper lip white with some vertical black stripes anterior to eye; iris bronze; ventral surface of head creamy white with some indistinct dark stripes on lower lip.

Dorsal surface of body gradually from greyish brown to black posteriorly; dorsolateral stripes indistinct anterior and distinct posteriorly, light yellow intermixed with light orange spots from nape to anterior tail; venter white with grayish black spots on edges of each ventral.

Dorsal surface of tail black, ventral surface of tail grayish white.

##### Variation.

The paratypes are very similar to the holotype in morphological characters (Table 3). The total length varies from 506 mm to 610 mm, the number of ventrals varies from 149 to 155, the number of subcaudals varies from 99 to 105, and the number of maxillary teeth varies from 23 (22+1) to 25 (23+2) in the paratypes.

**Table 3.** Morphometrics and meristics of the type series of *Hebius shantianfangi* sp. nov. For abbreviations see Material and methods.

	KIZ 064970 Holotype ♀	KIZ 064971 Paratype ♂	KIZ 064972 Paratype ♀	KIZ 064973 Paratype ♀
SVL	371	332	406	388
TaL	168	174	204	189
TL	539	506	610	577
HL	16.5	15.1	17.8	16.4
SnL	3.9	3.5	4.2	4.0
EDH	2.8	2.6	3.1	2.9
EDV	2.6	2.2	3.0	2.5
DLip	1.1	1.1	1.5	1.2
DS	19-19-17	19-19-17	19-19-17	19-19-17
Ven	148	155	149	155
Sc	90	105	99	100
SL	9/10	9/9	9/9	9/9
IL	10/10	10/10	11/11	9/9
PreOc	1/1	1/1	1/1	1/1
PosOc	3/3	3/3	3/3	3/3
Atem	1/1	1/1	1/1	1/1
MXT	24+1	22+2	23+2	22+1

##### Comparisons.

*Hebius shantianfangi* sp. nov. can be easily distinguished from all other species of the genus, except *H. boulengeri*, in having a distinct continuous white streak from the posteroventral margin of the eye extending backward and upward to the nape and connecting with the dorsolateral stripe on each side.

*Hebius shantianfangi* sp. nov. further differs from *H. annamensis* (Bourret, 1934), *H. arquus* (David & Vogel, 2010), *H. atemporalis* (Bourret, 1934), *H. celebicus* (Peters & Doris, 1878), *H. chapaensis* (Bourret, 1934), *H. citrinoventer* Xu, Yang, Gong, Ouyang, Weng, Deng, Huang & Peng, 2024, *H. frenatus* (Dunn, 1923), *H. groundwateri* (Smith, 1922), *H. maximus* (Malnate, 1962), *H. nigriventer* (Wall, 1925), *H. sarasinorum* (Boulenger, 1896), *H. sarwacensis* (Günther, 1872), *H. sauteri* (Boulenger, 1909), *H. taronensis* (Smith, 1940), *H. venningi* (Wall, 1910), and *H. youjiangensis* Yang, Xu, Wu, Gong, Huang & Huang, 2023 in having 19 dorsal scale rows at midbody (vs 15 or 17 rows at midbody).

*Hebius shantianfangi* sp. nov. further differs from *H. annamensis*, *H. chapaensis*, *H. deschauenseei* (Taylor, 1934), *H. igneus* David, Vogel, Nguyen, Orlov, Pauwels, Teynié & Ziegler, 2021, *H. modestus* (Günther, 1875), *H. nigriventer*, *H. taronensis*, *H. venningi*, and *H. youjiangensis* in having a white venter (vs dark venter).

*Hebius shantianfangi* sp. nov. further differs from *H. andreae* (Ziegler & Le Khac Quyet, 2006), *H. arquus*, *H. beddomei* (Günther, 1864), *H. celebicus*, *H. concelarus* (Malnate, 1963), *H. flavifrons* (Boulenger, 1887), *H. frenatus*, *H. groundwateri*, *H. ishigakiensis* (Malnate & Munsterman, 1960), *H. lacrima* Purkayastha & David, 2019, *H. nicobariensis* (Sclater, 1891), *H. optatus* (Hu & Zhao, 1966), *H. petersii* (Boulenger, 1893), *H. pryeri* (Boulenger, 1887), *H. sanguineus* (Smedley, 1932), *H. sarasinorum*, *H. sarwacensis*, *H. vibakari* (Boie, 1826), and *H. yanbianensis* Liu, Zhong, Wang, Liu & Guo, 2018 in having dorsolateral stripes (vs no dorsolateral stripes but blotches or crossbars).

*Hebius shantianfangi* sp. nov. further differs from *H. bitaeniatus* (Wall, 1925) in having more subcaudals (90–105 vs 76–86), further differs from *H. clerki* (Wall, 1925) in having fewer ventrals (148–155 vs 162–173), further differs from *H. craspedogaster* (Boulenger, 1899) in having unkeeled the 1^st^ dorsal scale row on each side (vs keeled the 1^st^ dorsal scale row), further differs from *H. inas* (Laidlaw, 1901) in having dark grey blotches on the edges of each ventral (vs dark reddish-brown on the outer quarter or third of each ventral on each side), further differs from *H. jingdongensis* Ma, Shi, Ayi & Jiang, 2023 in having fewer ventrals (148–155 vs 163–166), further differs from *H. johannis* (Boulenger, 1908) in having fewer ventrals (148–155 vs 165–170), further differs from *H. kerinciensis* (David & Das, 2003) in having more ventrals (148–155 vs 138–140), further differs from *H. leucomystax* (David, Bain, Quang Truong, Orlov, Vogel, Ngoc Thanh & Ziegler, 2007) in having a white streak from the posteroventral margin of the eye extending to the nape on each side (vs a white streak from the tip of the snout extending under the eye to the nape on each side), further differs from *H. metusia* (Inger, Zhao, Shaffer & Wu, 1990) in having fewer ventrals (148–155 vs 159–176), further differs from *H. miyajimae* (Maki, 1931) in having more supralabials (9–10 vs 8), further differs from *H. octolineatus* (Boulenger, 1904) in having more subcaudals (90–105 vs 70–80), further differs from *H. parallelus* (Boulenger, 1890) in having fewer ventrals (148–155 vs 160–173), further differs from *H. popei* (Schmidt, 1925) in having more ventrals (148–155 vs 130–142), further differs from *H. sangzhiensis* Zhou, Qi, Lu, Lyu & Li, 2019 in having fewer ventrals (148–155 vs 160–164), further differs from *H. septemlineatus* (Schmidt, 1925) in having fewer ventrals (148–155 vs 164–175), further differs from *H. terrakarenorum* Hauser, Smits & David, 2022 in having fewer ventrals (148–155 vs 159–171), further differs from *H. viperinus* (Schenkel, 1901) in having more ventrals (148–155 vs 101), and further differs from *H. weixiensis* Hou, Yuan, Wei, Zhao, Liu, Wu, Shen, Chen, Guo & Che, 2021 in having fewer ventrals (148–155 vs 171–182).

*Hebius shantianfangi* sp. nov. is closely related to *H. khasiensis*, *H. gilhodesi*, and *H. boulengeri* according to the molecular phylogeny. However, *Hebius shantianfangi* sp. nov. can be easily distinguished from *H. khasiensis* and *H. gilhodesi* in having a continuous white streak from the posteroventral margin of the eye extending to the nape on each side (vs a discontinuous white streak or rounded blotches on each side). In addition, the dorsolateral stripes are light yellow intermixed with light orange spots in *Hebius shantianfangi* sp. nov., whereas the dorsolateral stripes are rusty brown or burnt sienna intermixed with yellowish-ochre spots in *H. khasiensis* and absent or very indistinct composed of obscure, tiny faded or ochre spots in *H. gilhodesi*, and *Hebius shantianfangi* sp. nov. has 23–25 maxillary teeth whereas *H. khasiensis* has 17–21 and *H. gilhodesi* has 21–23 maxillary teeth.

*Hebius shantianfangi* sp. nov. superficially closely resembles and has the smallest genetic distance from *H. boulengeri* phylogenetically. Based on our morphological data obtained from specimen examination (Suppl. material 1), *Hebius shantianfangi* sp. nov. differs from the type specimens of *H. boulengeri* (*n* = 2) in having more ventrals (148–155 vs 139–140), more postoculars (3 vs 2), relatively larger eyes (EDH/SnL 0.72–0.74 vs 0.66–0.69, EDV/DLip 2.00–2.36 vs 1.41–1.69), and fewer maxillary teeth (23–25 vs 27). *Hebius shantianfangi* sp. nov. differs from the other specimens from Guangdong Province, China (*n* = 2), in having more ventrals (148–155 vs 141–146), more postoculars (3 vs 2), relatively larger eyes (EDH/SnL 0.72–0.74 vs 0.68, EDV/DLip 2.00–2.36 vs 1.93), and fewer maxillary teeth (23–25 vs 26). *Hebius shantianfangi* sp. nov. differs from the specimens from Guangxi Autonomous Region and Guizhou Province, China (*n* = 2), in having a relatively shorter snout (SnL/HL 0.23–0.24 vs 0.25) and relatively larger eyes (EDH/SnL 0.72–0.74 vs 0.67, EDV/DLip 2.00–2.36 vs 1.67). *Hebius shantianfangi* sp. nov. differs from the specimens from Vietnam and Cambodia (*n* = 71) in having fewer maxillary teeth (23–25 vs 26–28). *Hebius shantianfangi* sp. nov. differs from the specimens from Laos (*n* = 2) in having more ventrals (148–155 vs 142–144), a relatively shorter tail (TaL/TL 0.31–0.34 vs 0.35–0.36), a relatively shorter head (HL/SVL 0.042–0.045 vs 0.053–0.054), a relatively shorter snout (SnL/HL 0.23–0.24 vs 0.25–0.26), and fewer maxillary teeth (23–25 vs 26). *Hebius shantianfangi* sp. nov. differs from the specimens from Thailand (*n* = 2) in having a relatively shorter head (HL/SVL 0.042–0.045 vs 0.047) and a relatively smaller distance between the lower edge of the lip and the lower margin of the eye (EDV/DLip 2.00–2.36 vs 1.81–1.94). In addition, the edges of the white streak posterior to the eye are jagged and bordered by indistinct black color and the dorsolateral stripes are light yellow intermixed with light orange spots in *Hebius shantianfangi* sp. nov., whereas the edges of the white streak posterior to the eye are smooth and bordered by distinct black color and the dorsolateral stripes are orange intermixed with red spots in *H. boulengeri* from Guangdong Province, China (Figs 5, 6).

##### Distribution.

**Figure 7.**
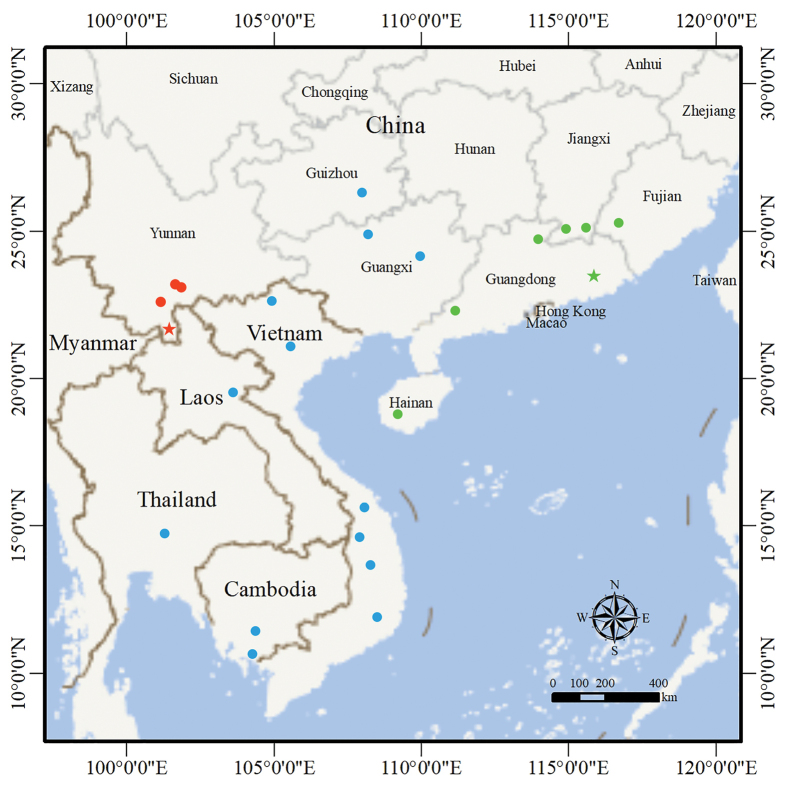
Map showing the distributions of *Hebius shantianfangi* sp. nov. (red), *H. boulengeri* sensu stricto (green), and Hebius cf. boulengeri (blue). Star indicates type locality.

The new species is currently known only from Xishuangbanna Prefecture and Pu’er City, Yunnan Province, China (Fig. 7).

##### Ecology notes.

The specimens of the new species were discovered on the ground beside streams at night. Additional ecological information about this species is unknown. Other species of amphibians and reptiles found at the type locality include *Amolops vitreus* (Bain, Stuart & Orlov, 2006), *Hylarana menglaensis* Fei, Ye & Xie, 2008, *Kurixalus odontotarsus* (Ye & Fei, 1993), *Limnonectes bannaensis* Ye, Fei & Jiang, 2007, *L. limborgi* (Sclater, 1892), *Leptobrachella eos* (Ohler, Wollenberg, Grosjean, Hendrix, Vences, Ziegler & Dubois, 2011), *L. ventripunctata* (Fei, Ye & Li, 1990), *Microhyla mukhlesuri* Hasan, Islam, Kuramoto, Kurabayashi & Sumida, 2014, *Polypedates impresus* Yang, 2008, *Zhangixalus pachyproctus* Yu, Hui, Hou, Wu, Rao & Yang, 2019, *Acanthosaura rubrilabris* Liu, Rao, Hou, Orlov, Ananjeva & Zhang, 2022, *Calotes emma* Gray, 1845, *Pareas berdmorei* Theobald, 1868, and *Trimeresurus lanna* Idiiatullina, Nguyen, Pawangkhanant, Suwannapoom, Chanhome, Mirza, David, Vogel & Poyarkov, 2024.

## ﻿Discussion

Through morphological and molecular data, this study demonstrated that the populations from Mengla County of Xishuangbanna Prefecture and Mojiang County of Pu’er City, Yunnan Province, which were previously considered as *H. boulengeri*, represent a distinct species, which we described herein as new. In addition, *H. boulengeri* was also considered to be distributed in other areas of Yunnan Province, such as Hekou, Lvchun, and Pingbian counties of Honghe Prefecture, Menglian County of Pu’er City, Cangyuan County of Lincang City, Longling County of Baoshan City, Gongshan County of Nujiang Prefecture, and Weixi County of Diqing Prefecture (Yang and Rao 2008). For the populations that were identified as *H. boulengeri* in these areas of Yunnan Province, we speculate that they also do not belong to *H. boulengeri* sensu stricto. Due to the relatively conservative morphology and the lack of available molecular data for these populations, their taxonomic status remains to be determined.

Phylogenetic analysis revealed relatively significant genetic differentiation within the populations currently referred to as *Hebius boulengeri* (Fig. 1). The sequences of specimens from Guangdong (KJ685684, MK201380, and MK201509), Fujian (KJ685699), Jiangxi (MK201483), and Hainan (KJ685691, KJ685710, and MK201295) provinces of China clustered together to form a less divergence clade, with 1.8% genetic distance within it. The sequences of specimens from northwestern Guangxi Autonomous Region of China (MK201520) and northern Vietnam (KJ685664 and OK315817) formed two distinct clades, respectively, and there were relatively large genetic distances (3.3–4.4%) between them and the clade from Guangdong, Fujian, Jiangxi, and Hainan. In addition, the other two specimens from Guangdong we examined are in good agreement with the type specimens in morphology, while the specimens from Guangxi Autonomous Region and Guizhou Province of China, Vietnam, Cambodia, Laos, and Thailand, show some morphological differences (e.g. number of ventrals, subcaudals, and postoculars; Suppl. material 1) from the type specimens and from each other. Therefore, it is likely that *H. boulengeri* sensu stricto is restricted to Guangdong, Fujian, Jiangxi, and Hainan provinces, and Hong Kong Special Administrative Region, in China. The populations of *H. boulengeri* sensu lato from Guangxi and Guizhou of China, Vietnam, Cambodia, Laos, and Thailand, may represent multiple cryptic species, which are currently best referred to as Hebius cf. boulengeri. Further studies combining morphological and molecular data will reveal their taxonomic status.

## Supplementary Material

XML Treatment for Hebius shantianfangi

## ﻿Additional information

### Use of AI

No use of AI was reported.

### Author ORCIDs

Shuo Liu https://orcid.org/0000-0001-7825-3006

JiShan Wang https://orcid.org/0000-0003-4660-163X

Mian Hou https://orcid.org/0000-0002-3322-6570

Dingqi Rao https://orcid.org/0000-0003-2680-1503

Patrick David https://orcid.org/0000-0001-7562-684X

Gernot Vogel https://orcid.org/0000-0002-4542-518X

### Data availability

All of the data that support the findings of this study are available in the main text or Supplementary Information.

## ﻿Appendix 1

**Specimens examined**

*Hebius boulengeri* (*n* = 81): MVZ 23623, holotype, Jieyang, Guangdong, China; MVZ 23622, paratype, Ganzhou, Jiangxi, China; ZMB 27694, northern Guangdong, China; SMNH 1238, Guangdong, China; CIB 602339, Laibin, Guangxi, China; CIB 63II5098, Leishan, Guizhou, China; AMNH 148552, 148562, Ha Giang, Vietnam; IEBR 1290, MNHN 1997.3307, 1999.9093–1999.9094, 1935.0061–1935.0063, 1935.0451–1935.0452, 1935.0453, 1958.0459–1958.0460, PSGV 0002 S, MVZ 224141–224143, 224145–224148, 224153, 226513, MVZ 226515, Tam Dao, Vinh Phu, Vietnam; CAS 73737, FMNH 71709, southern Vietnam; BMNH 1921.4.1.3–1921.4.1.5, 1969.1716–1969.1718, Lang Bian, Vietnam; FMNH 252117, 252120–252123, Ankhe, Gia Lai, Vietnam; IEBR 353, 1650–1652, 1654–1656, 1658–1662, 2540–2541, 2537, Kon Tum, Vietnam; IEBR 2977, ROM 03848, 21434, 21438, Tay Gian, Quang Nam, Vietnam; BMNH 1928.6.29.9, 1969.1710–1969.1715, 1969.1720, Bokor Mts., Cambodia; LSUHC 07442–07444, 07464–07465, 07484, Phnom Aural, Cambodia; MNHN 1928.0056–1928.0057, Xiengkhuang, Laos; FMNH 180153–180154, Khao Yai, Thailand.

## Supplementary materials

Supplementary material 1 Detailed measurements and scale counts for each examined specimen of *Hebius boulengeri* Data type xlsx Explanation note All measurements are in the unit of mm. “N/A” indicates not available. For measurement and count methods and abbreviations, see the Materials and methods. https://binary.pensoft.net/file/1429572This dataset is made available under the Open Database License (http://opendatacommons.org/licenses/odbl/1.0/). The Open Database License (ODbL) is a license agreement intended to allow users to freely share, modify, and use this Dataset while maintaining this same freedom for others, provided that the original source and author(s) are credited.Patrick David, Gernot Vogel
